# Supplementary material for: Mice Condition Cephalic-Phase Insulin Release to Flavors Associated with Postoral Actions of Concentrated Glucose
Source: Nutrients. 2024 Jul 12;16(14):2250. doi: 10.3390/nu16142250 (PMC11279997; doi:10.3390/nu16142250)
Supplement: Supplementary file 1 [file nutrients-16-02250-s001.zip › nutrients-3089084-supplementary.pdf]

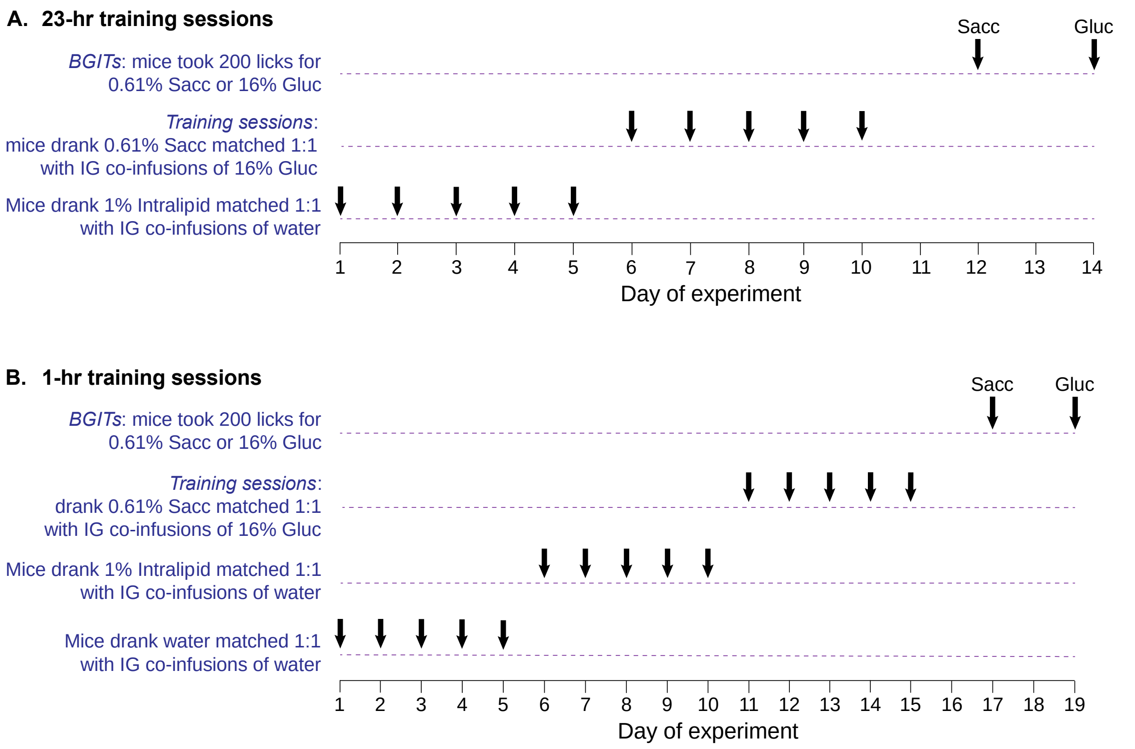


Figure S1. Timeline of the experimental interventions in Experiment 1. The interventions occurred during daily 23-hr (A) or 1-hr (B) training sessions.

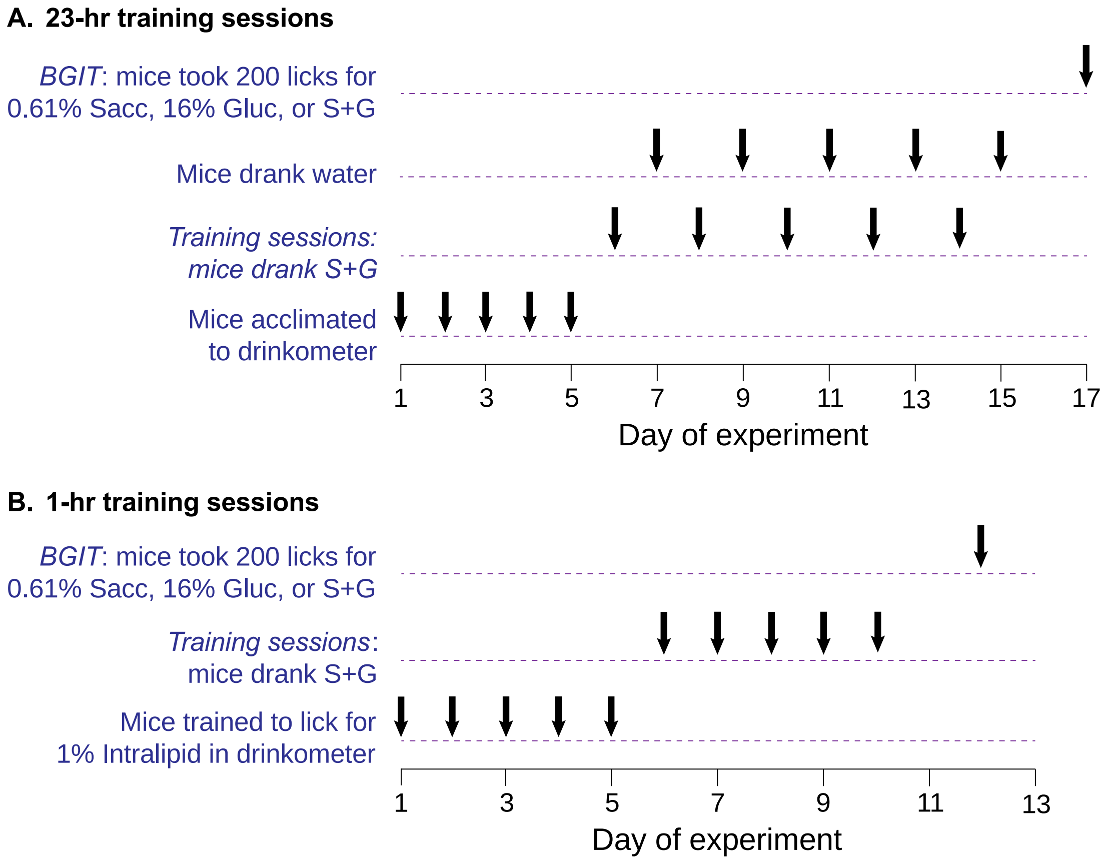


Figure S2. Timeline of the experimental interventions in Experiment 2. The interventions occurred during daily 23-hr (A) or 1-hr (B) training sessions.

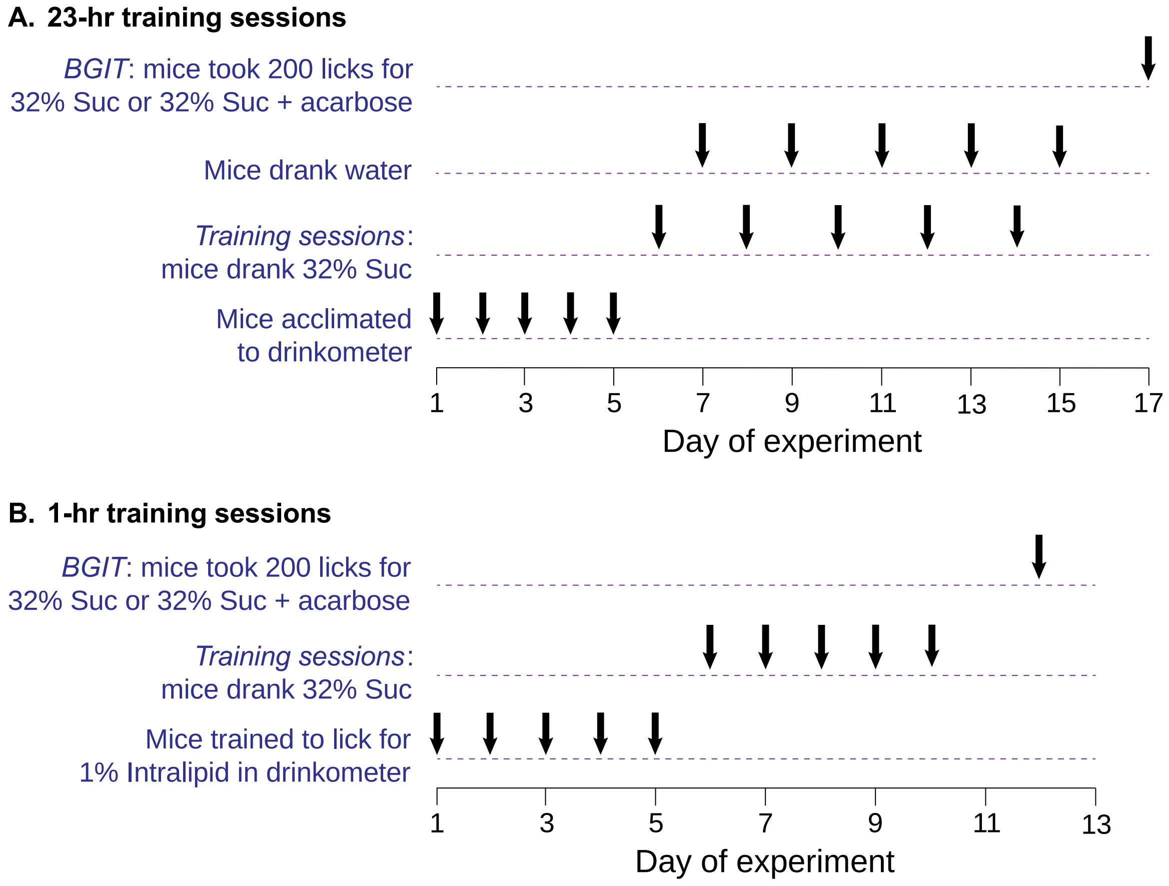


Figure S3. Timeline of the experimental interventions in Experiment 3. The interventions occurred during daily 23-hr (A) or 1-hr (B) training sessions.

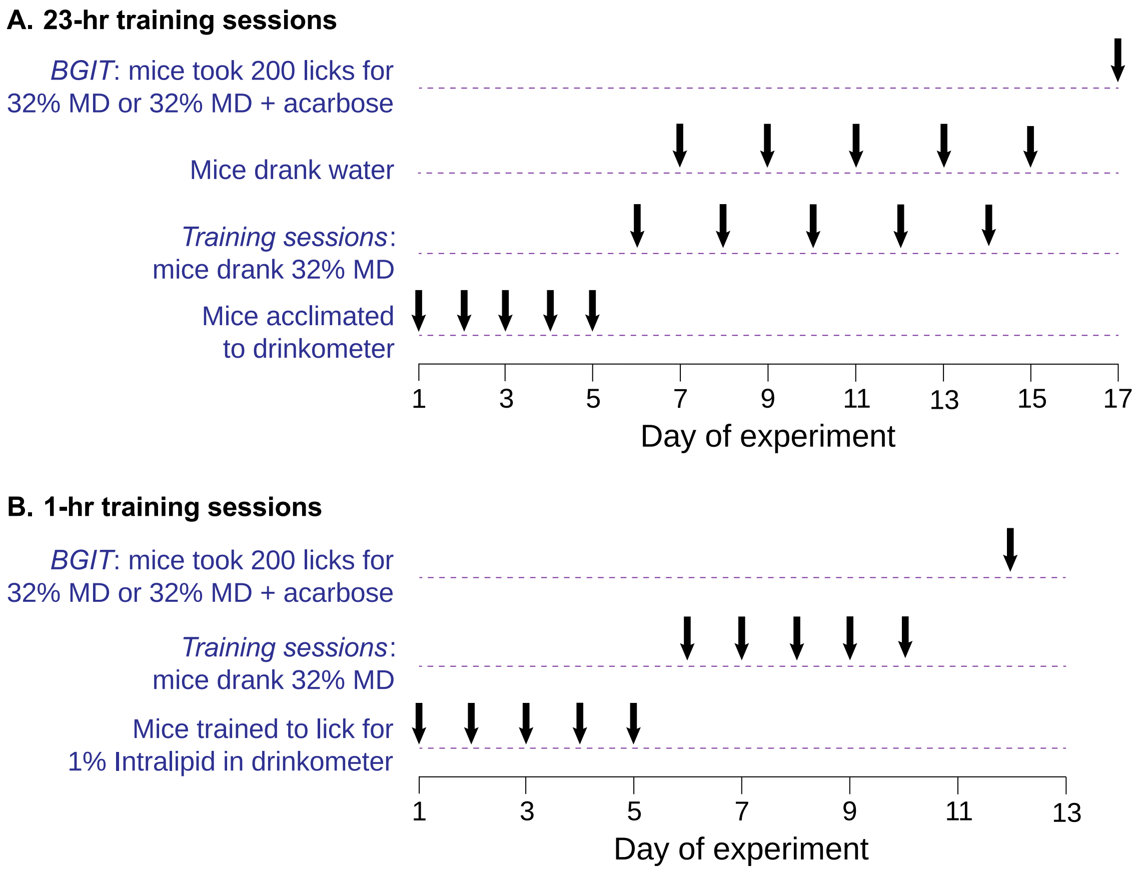


Figure S4. Timeline of the experimental interventions in Experiment 4. The interventions occurred during daily 23-hr (A) or 1-hr (B) training sessions.

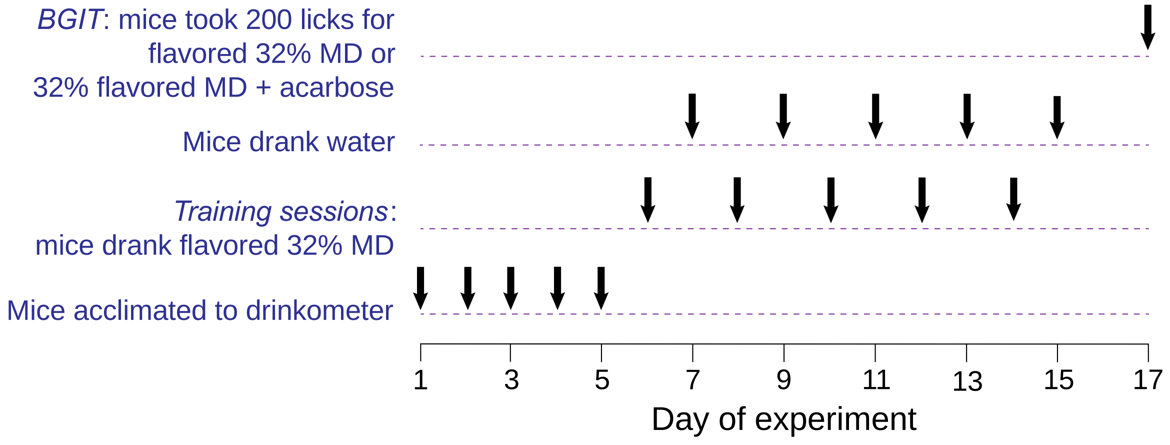


Figure S5. Timeline of the experimental interventions in Experiment 5. The interventions occurred during daily 23-hr training sessions.

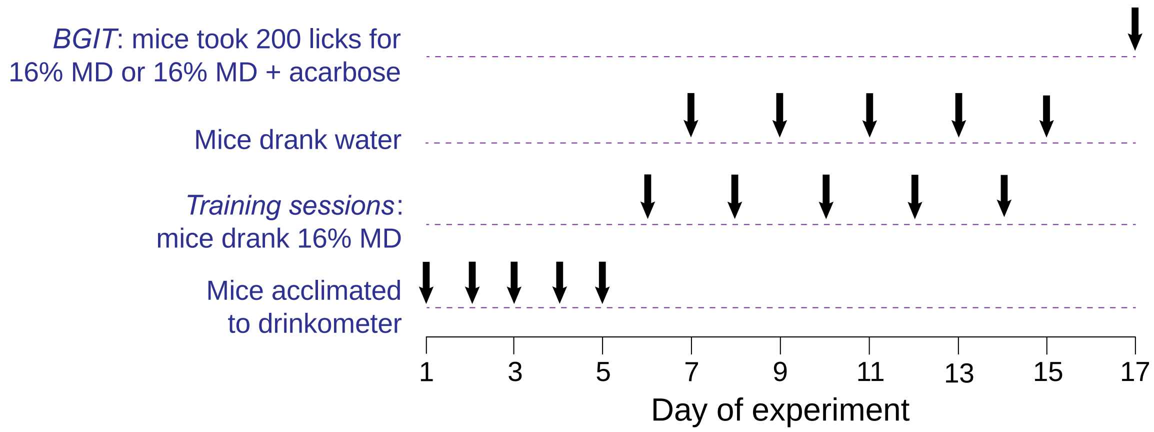


Figure S6. Timeline of the experimental interventions in Experiment 6. The interventions occurred during daily 23-hr training sessions.

Table S1. Analysis of the results in Fig. 1.

| Dependent measure | Training session duration | Source of variation | df | F-ratio | P-value |
|---|---|---|---|---|---|
| Plasma insulin levels across BGIT | 23-hr | Test solutions | 1, 4 | 16.9 | 0.015 |
| | | Time across BGIT | 1.2, 4.6 | 8.0 | 0.038 |
| | | Interaction | 1.4, 5.4 | 8.0 | 0.029 |
| | 1-hr | Test solutions | 1, 7 | 2.2 | 0.18 |
| | | Time across BGIT | 2.2, 15.5 | 10.6 | 0.001 |
| | | Interaction | 1.7, 12.0 | 8.5 | 0.007 |
| Blood glucose levels across BGIT | 23-hr | Test solutions | 1, 4 | 19.1 | 0.012 |
| | | Time across BGIT | 1.8, 7.4 | 12.7 | 0.004 |
| | | Interaction | 2.0, 8.0 | 9.6 | 0.007 |
| | 1-hr | Test solutions | 1, 7 | 140.3 | < 0.0001 |
| | | Time across BGIT | 3.1, 22.0 | 55.6 | < 0.0001 |
| | | Interaction | 2.7, 18.8 | 37.9 | < 0.0001 |

Prior to the BGITs, the mice were subjected to 23-hr or 1-hr training sessions, during which consumption of 0.61% Sacc was paired with IG co-infusions of 16% Gluc. Afterwards, all mice were run through two 60-min BGITs, one with 0.61% Sacc and the other with 16% Gluc. Plasma insulin and blood glucose levels were measured at 5 time-points across the BGIT. Each dependent measure was analyzed with a mixed-model ANOVA. The degrees of freedom were subjected to a Geisser-Greenhouse correction.

Table S2. Analysis of the results in Fig. 2.

| Dependent measure | Training sessions | Source of variation | df | F-ratio | P-value |
|---|---|---|---|---|---|
| Intake during the training sessions | 23-hr | Test solutions | 2, 21 | 0.2 | 0.78 |
| | | Training sessions | 1.8, 37.2 | 4.8 | 0.018 |
| | | Interaction | 8, 84 | 1.9 | 0.066 |
| | 1-hr | Test solutions | 2, 24 | 0.2 | 0.78 |
| | | Training sessions | 2.3, 56.[illegible] | 19.5 | <0.0001 |
| | | Interaction | 8, 96 | 1.4 | 0.19 |
| Plasma insulin levels across the BGIT | 23-hr | Test solutions | 2, 20 | 8.8 | 0.0018 |
| | | Time across BGIT | 3.3, 66.6 | 16.4 | < 0.0001 |
| | | Interaction | 8, 80 | 4.7 | 0.0001 |
| | 1-hr | Test solutions | 2, 24 | 5.4 | 0.012 |
| | | Time across BGIT | 2.5, 60.4 | 20.6 | < 0.0001 |
| | | Interaction | 8, 96 | 7.4 | < 0.0001 |
| | None | Test solutions | 2, 22 | 18.8 | < 0.0001 |
| | | Time across BGIT | 2.9, 63.0 | 41.8 | < 0.0001 |
| | | Interaction | 8, 88 | 14.9 | < 0.0001 |
| Blood glucose levels across the BGIT | 23-hr | Test solutions | 2, 20 | 25.0 | < 0.0001 |
| | | Time across BGIT | 2.4.47.4 | 63.2 | < 0.0001 |
| | | Interaction | 8, 80 | 12.2 | < 0.0001 |
| | 1-hr | Test solutions | 2, 24 | 41.6 | < 0.0001 |
| | | Time across BGIT | 2.2, 52.5 | 48.8 | < 0.0001 |
| | | Interaction | 8, 96 | 12.1 | < 0.0001 |
| | None | Test solutions | 2, 22 | 16.5 | < 0.0001 |
| | | Time across BGIT | 2.6, 57.1 | 51.9 | < 0.0001 |
| | | Interaction | 8, 88 | 11.7 | < 0.0001 |

Prior to the BGIT, the experimental mice were subjected to 23-hr, 1-hr or no training sessions with the S+G solution (i.e., 0.61% Sacc + 16% Gluc). Subsequently, all mice were run through a 60-min BGIT with one of three test solutions: 0.61% Sacc, 16% Gluc, or S+G. Plasma insulin and blood glucose levels were measured at 5 time-points across the BGIT. Each dependent measure was analyzed with a mixed-model ANOVA. The degrees of freedom were subjected to a Geisser-Greenhouse correction.

Table S3. Analysis of the results in Fig 3.

| Dependent measure | Training sessions | Source of variation | df | F-ratio | P-value |
|---|---|---|---|---|---|
| Intake during the training sessions | 23-hr | Test solutions | 1, 18 | 0.1 | 0.71 |
| | | Training sessions | 2, 36 | 3.3 | 0.049 |
| | | Interaction | 4, 72 | 0.5 | 0.71 |
| | 1-hr | Test solutions | 1, 14 | < 0.1 | 0.87 |
| | | Training sessions | 2.9, 41.0 | 13.4 | < 0.0001 |
| | | Interaction | 4, 56 | 0.5 | 0.71 |
| Plasma insulin levels across the BGIT | 23-hr | Test solutions | 1, 18 | 12.8 | 0.002 |
| | | Time across BGIT | 2.6, 45.9 | 11.2 | < 0.0001 |
| | | Interaction | 4, 72 | 12.6 | < 0.0001 |
| | 1-hr | Test solutions | 1, 14 | 47.1 | < 0.0001 |
| | | Time across BGIT | 2.7, 37.2 | 21.4 | < 0.0001 |
| | | Interaction | 4, 56 | 9.7 | < 0.0001 |
| | None | Test solutions | 1, 14 | 13.3 | 0.003 |
| | | Time across BGIT | 1.8, 25.7 | 9.0 | 0.001 |
| | | Interaction | 4, 56 | 10.8 | < 0.0001 |
| Blood glucose levels across the BGIT | 23-hr | Test solutions | 1, 18 | 18.7 | 0.0004 |
| | | Time across BGIT | 2.8, 49.6 | 45.8 | < 0.0001 |
| | | Interaction | 4, 72 | 14.7 | < 0.0001 |
| | 1-hr | Test solutions | 1, 14 | 25.7 | 0.0002 |
| | | Time across BGIT | 1.5, 20.7 | 37.3 | < 0.0001 |
| | | Interaction | 4, 56 | 27.2 | < 0.0001 |
| | None | Test solutions | 1, 14 | 43.4 | < 0.0001 |
| | | Time across BGIT | 1.4, 19.2 | 21.8 | < 0.0001 |
| | | Interaction | 4, 56 | 13.7 | < 0.0001 |

Prior to the BGIT, the experimental mice were subjected to 23-hr, 1-hr or no training sessions with 32% Suc. Subsequently, all mice were run through a 60-min BGIT with one of two test solutions: 32% Suc + acarbose or 32% Suc. Plasma insulin and blood glucose levels were measured at 5 time-points across the BGIT. Each dependent measure was analyzed with a mixed-model ANOVA. The degrees of freedom were subjected to a Geisser-Greenhouse correction.

Table S4. Analysis of the results in Fig 4.

| Dependent measure | Training sessions | Source of variation | df | F-ratio | P-value |
|---|---|---|---|---|---|
| Intake during the training sessions | 23-hr | Test solutions | 1, 15 | 0.4 | 0.53 |
| | | Training sessions | 2.8, 41.3 | 2.5 | 0.079 |
| | | Interaction | 4, 60 | 2.1 | 0.091 |
| | 1-hr | Test solutions | 1, 17 | 0.5 | 0.49 |
| | | Training sessions | 2.5, 43.0 | 3.6 | 0.027 |
| | | Interaction | 4, 68 | 0.3 | 0.86 |
| Plasma insulin levels across the BGIT | 23-hr | Test solutions | 1, 15 | 3.7 | 0.075 |
| | | Time across BGIT | 2.9, 43.1 | 20.7 | < 0.001 |
| | | Interaction | 4, 60 | 4.2 | 0.004 |
| | 1-hr | Test solutions | 1, 28 | 4.8 | < 0.001 |
| | | Time across BGIT | 3.0, 82.6 | 36.2 | < 0.001 |
| | | Interaction | 4, 112 | 22.6 | < 0.001 |
| | None | Test solutions | 1, 15 | 15.9 | < 0.002 |
| | | Time across BGIT | 2.5, 38.4 | 54.8 | < 0.0001 |
| | | Interaction | 4, 60 | 40.6 | < 0.0001 |
| Blood glucose levels across the BGIT | 23-hr | Test solutions | 1, 15 | 14.6 | 0.002 |
| | | Time across BGIT | 2.4, 35.8 | 37.2 | < 0.001 |
| | | Interaction | 4, 60 | 4.5 | 0.003 |
| | 1-hr | Test solutions | 1, 28 | 38.2 | < 0.001 |
| | | Time across BGIT | 2.6, 73 | 54.4 | < 0.001 |
| | | Interaction | 4, 112 | 23.2 | < 0.001 |
| | None | Test solutions | 1, 14 | 29.3 | < 0.001 |
| | | Time across BGIT | 2.5, 34.6 | 55.6 | < 0.001 |
| | | Interaction | 4, 56 | 17.7 | < 0.001 |

Prior to the BGIT, the experimental mice were subjected to 23-hr, 1-hr, or no training sessions with 32% MD. Afterwards, all mice were run through a 60-min BGIT with one of two test solutions: 32% MD + acarbose or 32% MD. Plasma insulin and blood glucose levels were measured at 5 time-points across the BGIT. Each dependent measure was analyzed with a mixed-model ANOVA. The degrees of freedom were subjected to a Geisser-Greenhouse correction.

Table S5. Analysis of the results in Fig. 5.

| Dependent measure | Source of variation | df | F-ratio | P-value |
|---|---|---|---|---|
| Intake during the training sessions | Test solutions | 1, 17 | < 0.1 | 0.86 |
| | Training sessions | 3.0, 51.4 | 8.0 | 0.0002 |
| | Interaction | 4, 68 | 1.1 | 0.38 |
| Plasma insulin levels across the BGIT | Test solutions | 1, 17 | 11.7 | 0.003 |
| | Time across BGIT | 2.5, 42.8 | 39.2 | <0.0001 |
| | Interaction | 4, 68 | 21.7 | <0.0001 |
| Blood glucose levels across the BGIT | Test solutions | 1, 17 | 31.3 | < 0.0001 |
| | Time across BGIT | 2.3, 39.6 | 30.5 | < 0.0001 |
| | Interaction | 4, 68 | 6.7 | 0.0001 |

Prior to the BGIT, the mice were subjected to 23-hr training sessions with flavored 32% MD. Subsequently, all mice were run through a 60-min BGIT with one of two test solutions: flavored 32% MD + acarbose or flavored 32% MD. Plasma insulin and blood glucose levels were measured at 5 time-points across the BGIT. Each dependent measure was analyzed with a mixed-model ANOVA. The degrees of freedom were subjected to a Geisser-Greenhouse correction.

Table S6. Analysis of the results in Fig. 6.

| Dependent measure | Training sessions | Source of variation | df | F-ratio | P-value |
|---|---|---|---|---|---|
| Plasma insulin levels across BGIT | 23-hr | Test solutions | 1, 14 | 8.2 | < 0.013 |
| | | Time across BGIT | 2.4, 33.2 | 9.4 | < 0.0004 |
| | | Interaction | 4, 56 | 7.5 | < 0.0001 |
| | None | Test solutions | 1, 14 | 8.9 | < 0.01 |
| | | Time across BGIT | 2.2, 31.3 | 5.9 | < 0.006 |
| | | Interaction | 4, 56 | 11.7 | < 0.0001 |
| Blood glucose levels across BGIT | 23-hr | Test solutions | 1, 14 | 41.0 | < 0.0001 |
| | | Time across BGIT | 2.9, 40.4 | 38.3 | < 0.0001 |
| | | Interaction | 4, 56 | 13.1 | < 0.0001 |
| | None | Test solutions | 1, 14 | 33.8 | < 0.0001 |
| | | Time across BGIT | 3.1, 43.8 | 31.6 | < 0.0001 |
| | | Interaction | 4, 56 | 19.2 | < 0.0001 |

Prior to the BGIT, the experimental mice were subjected to 23-hr or no training sessions with 16% MD. Subsequently, all mice were run through a 60-min BGIT with one of two test solutions: 16% MD + acarbose or 16% MD. Plasma insulin and blood glucose levels were measured at 5 time-points across the BGIT. Each dependent measure was analyzed with a mixed-model ANOVA. The degrees of freedom were subjected to a Geisser-Greenhouse correction.
